# 397. The Three COVID-19 Prognostic Tools (CALL, 4Cs, NLR): Head-to-Head Comparison

**DOI:** 10.1093/ofid/ofad500.467

**Published:** 2023-11-27

**Authors:** George Doumat, Hady Yazbeck, Hassan Itani, Rayyan Wazzi-Mkahal, Mario Badr, Elie Moussa, Salim Barakat, Wael Osman, Zeina Kanafani

**Affiliations:** American University of Beirut, Beirut, Beyrouth, Lebanon; American University of Beirut, Beirut, Beyrouth, Lebanon; American University of Beirut, Beirut, Beyrouth, Lebanon; American University of Beirut, Beirut, Beyrouth, Lebanon; American University of Beirut, Beirut, Beyrouth, Lebanon; American University of Beirut, Beirut, Beyrouth, Lebanon; American University of Beirut, Beirut, Beyrouth, Lebanon; American University of Beirut, Beirut, Beyrouth, Lebanon; American University of Beirut Medical Center, Beirut, Beyrouth, Lebanon

## Abstract

**Background:**

Risk stratification of patients is crucial for treating COVID-19 infection, but studies have not compared prognostic tools like neutrophil to lymphocyte ratio (NLR), CALL score, and 4C mortality score. Our aim is to determine the most accurate prognostic score for predicting COVID-19 patient outcomes in a retrospective cohort at a tertiary care center in Lebanon.

**Methods:**

The study included adult patients with COVID-19 infection admitted from 2020 to 2021 at the American University of Beirut Medical Center. We calculated the NLR, CALL, and 4C scores and we recorded various outcomes for each patient. The outcomes of interest were need for mechanical ventilation (MV), intensive care unit (ICU) admission, illness progression, mortality, length of hospital stay, length of ICU stay, and length of MV. We also created a combined score from variables within each score and correlated it with outcome measures.

**Results:**

We enrolled 401 patients with confirmed COVID-19 infection. Patients were predominantly males (67%) with an average age of 67 years. The median length of hospital stay was 15 days and illness progression was recorded in 66% of patients. Around 50% of patients required ICU admission with a median stay of 14 days, and 31% required MV with a median duration of 16 days. The mortality rate was 31%. The CALL score was a significant predictor of need for ICU admission (difference of means [DOM] 0.73), MV (DOM 0.81), disease progression (DOM 0.78), and mortality (DOM 0.90), with p≤0.01 for each outcome). The 4C score was positively associated with disease progression (DOM 1.0) and mortality (DOM 1.9) (p≤0.03 for each outcome), while NLR was only predictive of disease progression (DOM 0.2; p=0.04). The combined score was able to predict mortality (DOM 1.7; p< 0.002) but none of the other outcomes.

**Conclusion:**

The CALL score proves to be the most useful tool in predicting several patient outcomes. However, the 4C score is a better predictor of mortality. There seems to be no added benefit from using a combined score. The evaluation of more advanced scoring systems is highly recommended to optimize risk stratification.

**Disclosures:**

**All Authors**: No reported disclosures

